# Resveratrol Improves Survival, Hemodynamics and Energetics in a Rat Model of Hypertension Leading to Heart Failure

**DOI:** 10.1371/journal.pone.0026391

**Published:** 2011-10-18

**Authors:** Stéphanie Rimbaud, Matthieu Ruiz, Jérôme Piquereau, Philippe Mateo, Dominique Fortin, Vladimir Veksler, Anne Garnier, Renée Ventura-Clapier

**Affiliations:** 1 UMR-S 769 Inserm, Univ Paris-Sud Châtenay-Malabry, Châtenay-Malabry, France; 2 Univ Paris-Sud, IFR 141, Châtenay-Malabry, France; Cardiovascular Research Institute Maastricht, Maastricht University, Netherlands

## Abstract

Heart failure (HF) is characterized by contractile dysfunction associated with altered energy metabolism. This study was aimed at determining whether resveratrol, a polyphenol known to activate energy metabolism, could be beneficial as a metabolic therapy of HF. Survival, ventricular and vascular function as well as cardiac and skeletal muscle energy metabolism were assessed in a hypertensive model of HF, the Dahl salt-sensitive rat fed with a high-salt diet (HS-NT). Resveratrol (18 mg/kg/day; HS-RSV) was given for 8 weeks after hypertension and cardiac hypertrophy were established (which occurred 3 weeks after salt addition). Resveratrol treatment improved survival (64% in HS-RSV versus 15% in HS-NT, p<0.001), and prevented the 25% reduction in body weight in HS-NT (P<0.001). Moreover, RSV counteracted the development of cardiac dysfunction (fractional shortening −34% in HS-NT) as evaluated by echocardiography, which occurred without regression of hypertension or hypertrophy. Moreover, aortic endothelial dysfunction present in HS-NT was prevented in resveratrol-treated rats. Resveratrol treatment tended to preserve mitochondrial mass and biogenesis and completely protected mitochondrial fatty acid oxidation and PPARα (peroxisome proliferator-activated receptor α) expression. We conclude that resveratrol treatment exerts beneficial protective effects on survival, endothelium–dependent smooth muscle relaxation and cardiac contractile and mitochondrial function, suggesting that resveratrol or metabolic activators could be a relevant therapy in hypertension-induced HF.

## Introduction

Heart failure (HF) is a complex and multicausal chronic syndrome characterized by profound myocardial dysfunction resulting in the inability of the cardiac pump to meet the requirements of the body. Alterations in energy metabolism are increasingly proposed as key events in the progression of the pathology [1]–[3] with effects also in peripheral muscles, thus suggesting a generalized metabolic myopathy in HF [4]–[6]. In addition, the phosphocreatine (PCr) to ATP ratio, a marker of energy metabolism of the myocardium has prognostic value in dilated cardiomyopathy [7]. These energetic alterations mainly originate from a down-regulation of genes involved in mitochondrial biogenesis and substrate utilization, the up-stream peroxisome proliferator-activated receptor (PPAR) gamma co-activator-1α (PGC-1α) and its downstream transcription factors and nuclear receptors [8], [9] (for review see [10]). Moreover, the decreased expression of PPARα in the failing heart leads to a marked reduction in fatty acid (FA) utilization [11]. It is thus largely accepted that a defective PGC-1α/PPARα axis plays a pivotal role in the pathogenesis of HF [12]. The generalized deficiency in energy metabolism, affecting substrate utilization, energy production, transfer and utilization in HF, thus paves the way for a metabolic therapy targeting energy starvation [2], [4].

Resveratrol, a polyphenol with antioxidant, anti-apoptotic, anti-inflammatory and metabolic properties was recently identified as an activator of energy metabolism. Enhanced SIRT1 activity decreases plasma glucose levels, improves insulin sensitivity, increases mitochondrial number and function, decreases adiposity, improves exercise tolerance and potentially lowers body weight [13]. Resveratrol can induce mitochondrial biogenesis and improve fatty acid oxidation (FAO) in many tissues via a mechanism involving sirtuins and AMP-activated protein kinase (AMPK) [14], [15]. In the heart, resveratrol decreases pressure overload-induced hypertrophy and contractile dysfunction [16]–[20]. A diet rich in polyphenols has been shown to reduce cardiovascular risk and induce vascular protection [21] and may exert anti-hypertensive and antiatherogenic effects [22]. Moreover resveratrol-rich extracts upregulate PGC-1α and PPARα cardiac expression and/or activity [23]. Because of its positive effects on vascular function and energy metabolism, we hypothesized that resveratrol could also have beneficial effects in HF. Therefore we examined in an experimental model, the Dahl-salt sensitive (DSS) rat, whether resveratrol is protective against the development of HF and associated metabolic dysfunction.

## Results

### Resveratrol improves survival and prevents body mass loss

**Figure 1:**
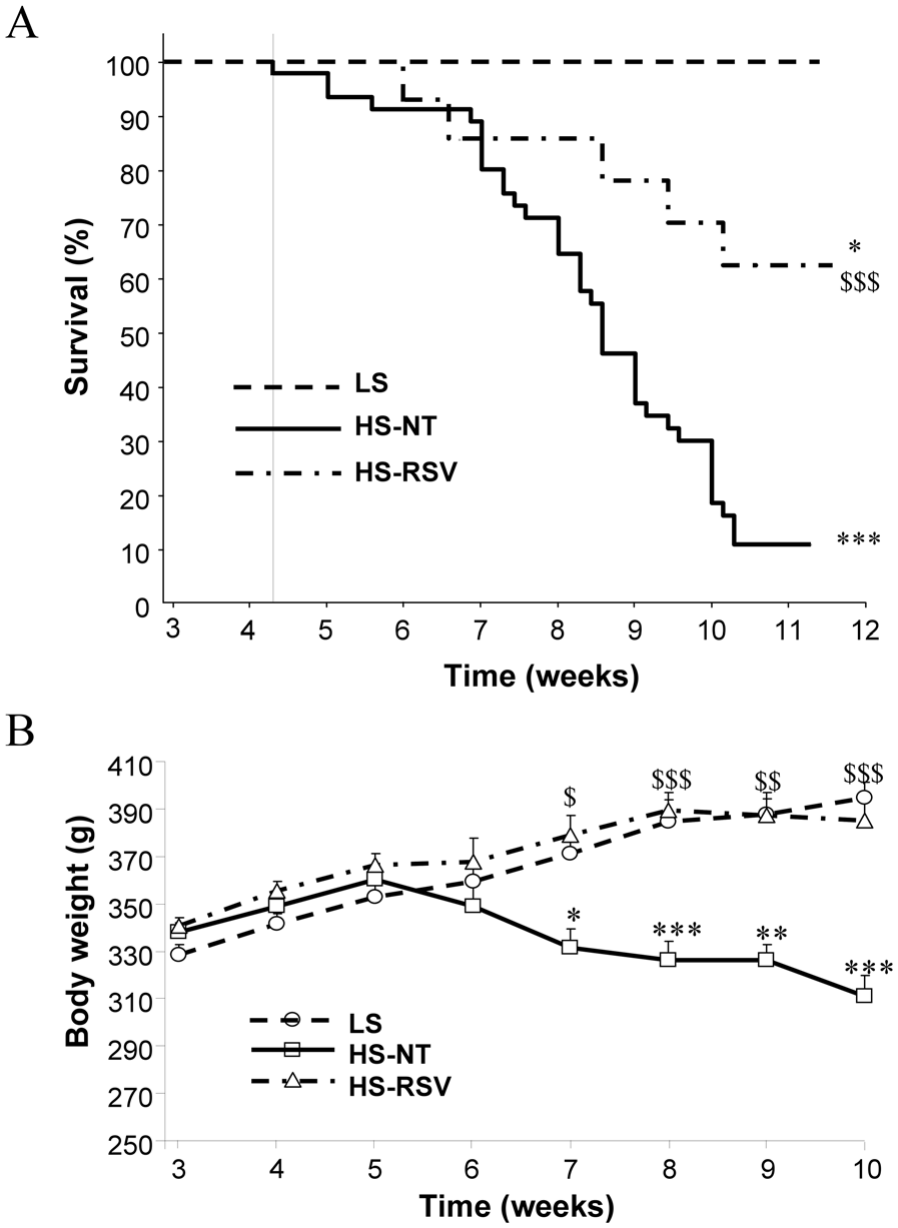
Resveratrol treatment increases survival and prevents body wasting in HS rats. (**A**) Kaplan-Meier survival analyses for low salt (LS, N = 9), high salt (HS, N = 48) and high salt treated with resveratrol (HS-RSV, N = 14) groups showed decreased survival of HS-NT compared to LS groups (log-rank: χ^2^ = 35.29, ***P<0.00001). Survival in HS-RSV rats was decreased compared to LS rats (log-rank: χ^2^ = 4.75, *P = 0.041) but was significantly higher than HS-NT rats (log-rank: χ^2^ = 39.78, ^$$$^P = 0.0007). **B.** The HS-NT group showed continuous weight loss from the fifth week of salt intake compared to LS. Resveratrol completely prevented HF-induced weight loss. *P<0.05, **P<0.01, ***P<0.001 vs LS; ^$^P<0.05, ^$$^P<0.01, ^$$$^P<0.001 vs HS-NT.

HS-NT animals had a low survival rate (7/48 of HS-NT rats, i.e. 14.6%), but this was largely increased with resveratrol treatment (9/14 of HS-RSV rats, i.e. 64.3%) (Figure 1A). Thus resveratrol treatment was strongly protective. At sacrifice, the body weight of HS-NT rats was 25% lower than that of both LS controls and HS-RSV rats (Figure 1B). Heart weight (absolute and normalized to tibia length) was significantly increased (by 33% and 35% respectively) in HS-NT animals (p<0.001) relative to LS animals and did not change with resveratrol treatment (Table 1). In HS-NT lung weight was increased and decreased by resveratrol only when related to body mass. The increase in kidney weight in HS-NT was moderately prevented by resveratrol treatment (Table 1). Total cholesterol, HDL and LDL were significantly increased in HS-NT compared to LS animals and tended to decrease with RSV treatment. No significant change in blood triglycerides and glucose was observed whatever the groups (Table S1).

**Table 1:** Structural and, functional parameters from LS, HS and RSV-treated rats.

	LS	HS-NT	HS-RSV
Anatomy	(N = 9)	(N = 7)	(N = 9)
**Body weight (BW, g)**	400±5	295±27***	393±14$$$
**Tibia length (TL, cm)**	4.49±0.02	4.39±0.05*	4.5±0.01$
**Heart weight (HW, g)**	1.35±0.03	1.79±0.11***	1.78±0.05***
**HW/BW (mg/g)**	3.4±0.1	6.3±0.6***	4.6±0.3* ^,^ $$
**HW/TL (mg/cm)**	301±7	406±23***	396±12***
**Lung W/TL (mg/cm)**	421±4	493±59	482±19
**LW/BW (mg/g)**	4.7±0.1	7.7±1.3*	5.6±0.4$
**Kidney W/TL (mg/cm)**	307±7	596±27***	501±33*** ^,^ $
**KW/BW (mg/g)**	3.4±0.1	9.2±0.7***	5.9±0.6**^,^ $$$

Echocardiography	(N = 8)	(N = 8)	(N = 9)
**IVSd (mm)**	1.61±0.10	2.06±0.12*	1.93±0.07*
**PWTd (mm)**	1.84±0.09	1.96±0.15	2.12±0.09
**Telediastolic volume (ml)**	0.91±0.04	1.23±0.16	0.99±0.08
**Telesystolic volume (ml)**	0.08±0.01	0.34±0.06***	0.14±0.04$$
**Ejection fraction (%)**	91.7±0.8	73.7±3.3***	87.4±2.2$$$
**Cardiac output (ml/min)**	311±13	241±26*	301±12$

Doppler analysis	(N = 5)	(N = 6)	(N = 9)
**Eann (m/s)**	0.082±0.01	0.046±0.003***	0.067±0.003*** ^,^ $$
**E/Eann**	14.2±1.9	22.0±3.2*	17.1±1.2

HW: heart weight; IVSd: interventricular septum in diastole; PWTd: posterior wall in diastole; TL: tibia length.

*P<0.05,

***P<0.001 vs LS;

$ P<0.05,

$$ P<0.01,

$$$ P<0.001 vs HS-NT.

### Resveratrol improves cardiac function and remodeling without changing blood pressure

**Figure 2:**
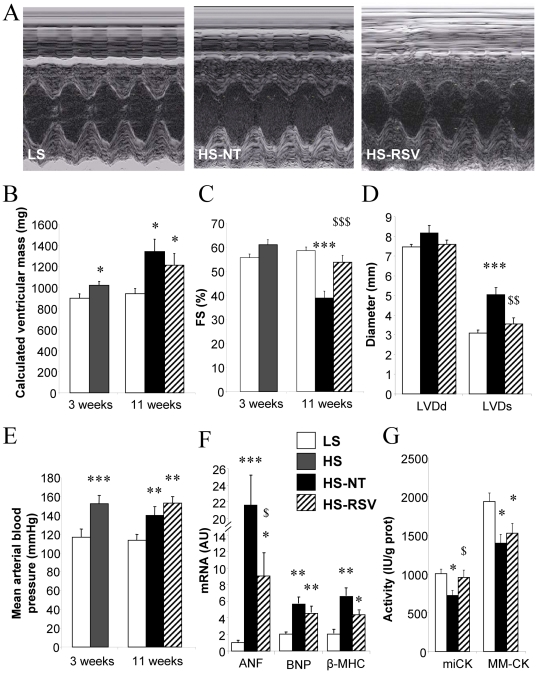
Resveratrol preserves cardiac function, with no effect on hypertrophy or hypertension. Cardiac morphology and function were assessed by echocardiography. (**A**) Representative M-mode images of the LV in LS, HS-NT and HS-RSV groups after 11 weeks of conditioning. (**B**) Calculated left ventricular mass and (**C**) fractional shortening (FS) at 3 and 11 weeks. (**D**) End-diastolic and end-systolic left ventricle diameter (LVDd and LVDs, respectively) at 11 weeks. (**E**) Mean arterial blood pressure. (**F**) Markers of remodeling: ANF (atrial natriuretic factor), BNP (brain natriuretic protein) β-MHC (β myosin heavy chain) and (**G**) energy metabolism miCK and MM-CK (mitochondrial and myofibrillar creatine kinase). Between 6 and 9 animals per group. *P<0.05, **P<0.01, ***P<0.001 vs LS; ^$^P<0.05, ^$$^P<0.01, ^$$$^P<0.001 vs HS-NT.

**Figure 3:**
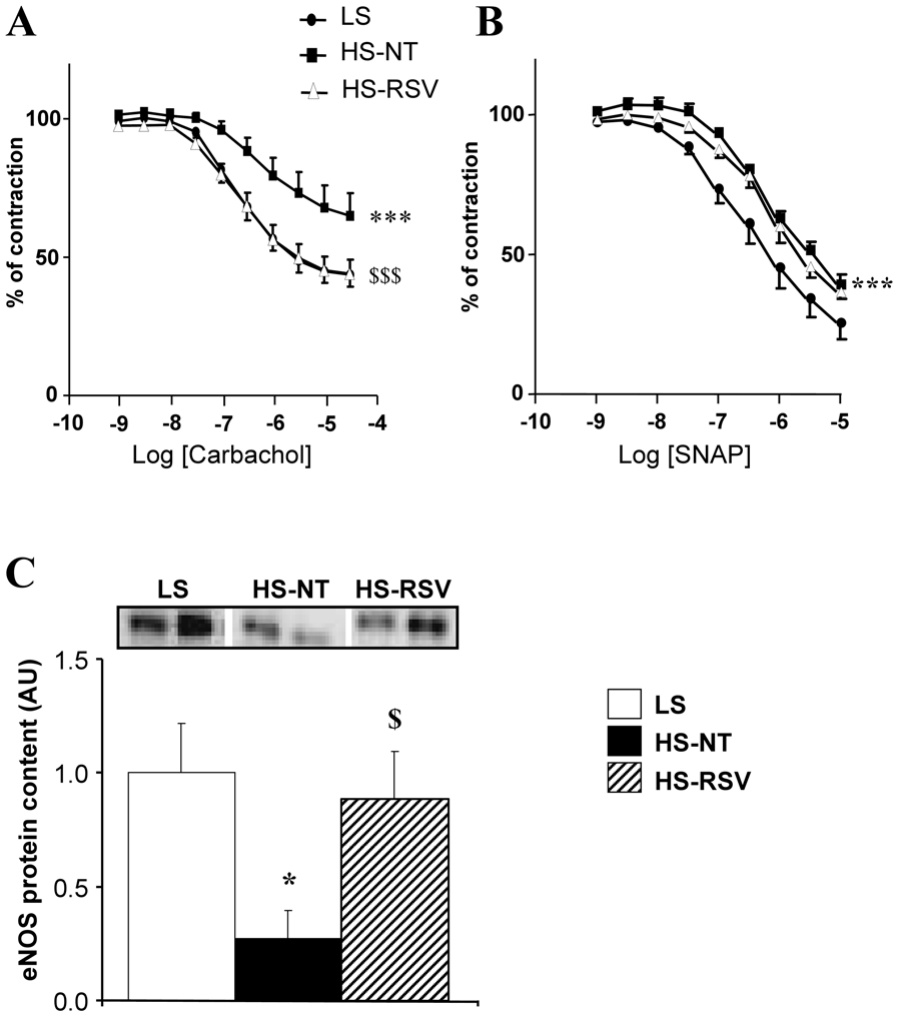
Endothelial function is preserved in aorta from resveratrol-treated rats. (**A**) Carbachol-induced relaxation of aortic rings precontracted with prostaglandin-F2α (PGF2α). (B) Endothelium-independent vasodilatation assessed using a NO donor (SNAP, S-nitrosothiol-N-acetyl-penicillamine) on PGF2α-preconstricted aorta rings. Between 6–8 experiments per group. (C) Western blot analysis of eNOS expression of LS (N = 4), HS-NT (N = 5) and HS-RSV (N = 7) aortas. *P<0.05, ***P<0.001 vs LS; ^$^P<0.05, ^$$$^P<0.001 HS-RSV vs HS-NT.

At 3 weeks, HS rat hearts had increased wall thickness (Table S2) and calculated ventricular mass compared with LS hearts (Figure 2B) with unaltered fractional shortening (FS, Figure 2C). Eight weeks later, ventricular mass was further increased both in HS-NT (+47%), and HS-RSV (+33%) compared to LS, with no significant difference between the two groups. HS-NT rats showed systolic dysfunction, with a 34% reduction in fractional shortening (FS, Figure 3C), 62% increase in end-systolic left ventricle (LV) diameter and 23% decrease in cardiac output, as well as diastolic dysfunction assessed by Doppler tissue imaging [24] (Table 1) whereas end-diastolic LV diameter was the same as in low salt controls (Figure 2D). No major change in intrinsic mechanical properties could be observed with the exception of the hill coefficient of calcium sensitivity (Table S3). Remarkably, both diastolic and systolic dysfunction was prevented by resveratrol treatment. Resting, maximal and mean blood pressure (BP) was already increased in HS compared with LS animals at 3 weeks (from 152±4 mmHg versus 116±9 mmHg, respectively), with no further increase occurring at 11 weeks in either HS-NT or HS-RSV groups (Figure 2E, Table S2). Thus resveratrol prevented cardiac dysfunction without affecting blood pressure and cardiac mass.

Expression of atrial natriuretic factor (ANF), brain natriuretic peptide (BNP) and myosin heavy chain β (β-MHC) mRNA, all markers of cardiac remodeling, was highly up-regulated in HS-NT animals, whereas in resveratrol-treated animals the increase in ANF was attenuated (Figure 2F). Similarly, in HS-NT animals there was a great decrease in mitochondrial and cytosolic creatine kinases (miCK and MM-CK) activities, markers of metabolic disturbances in HF, whereas mi-CK activity was maintained at the level of controls in HS-RSV animals (Figure 2G). The protein content of calcium metabolic markers like SERCA and calsequestrin did not significantly differ between these groups (Table S3).

### Resveratrol protects endothelial function

Carbachol-dependent relaxation of aorta after contraction induced by prostaglandin F2α was impaired in HS-NT animals compared with LS animals (Figure 3A). This impairment was completely prevented by resveratrol treatment. When nitric oxide (NO) was directly provided by S-nitrosothiol-N-acetyl-penicillamine (SNAP), a significant rightward shift was observed (p<0.001) in HS-NT rats that was not corrected by resveratrol (Figure 3B), suggesting an endothelium- and NO-dependent protective effect of resveratrol. Indeed, eNOS protein content was decreased in HS-NT and preserved by resveratrol treatment (Figure 3C).

### Resveratrol improves mitochondrial respiration, biogenesis and substrate utilization

**Figure 4:**
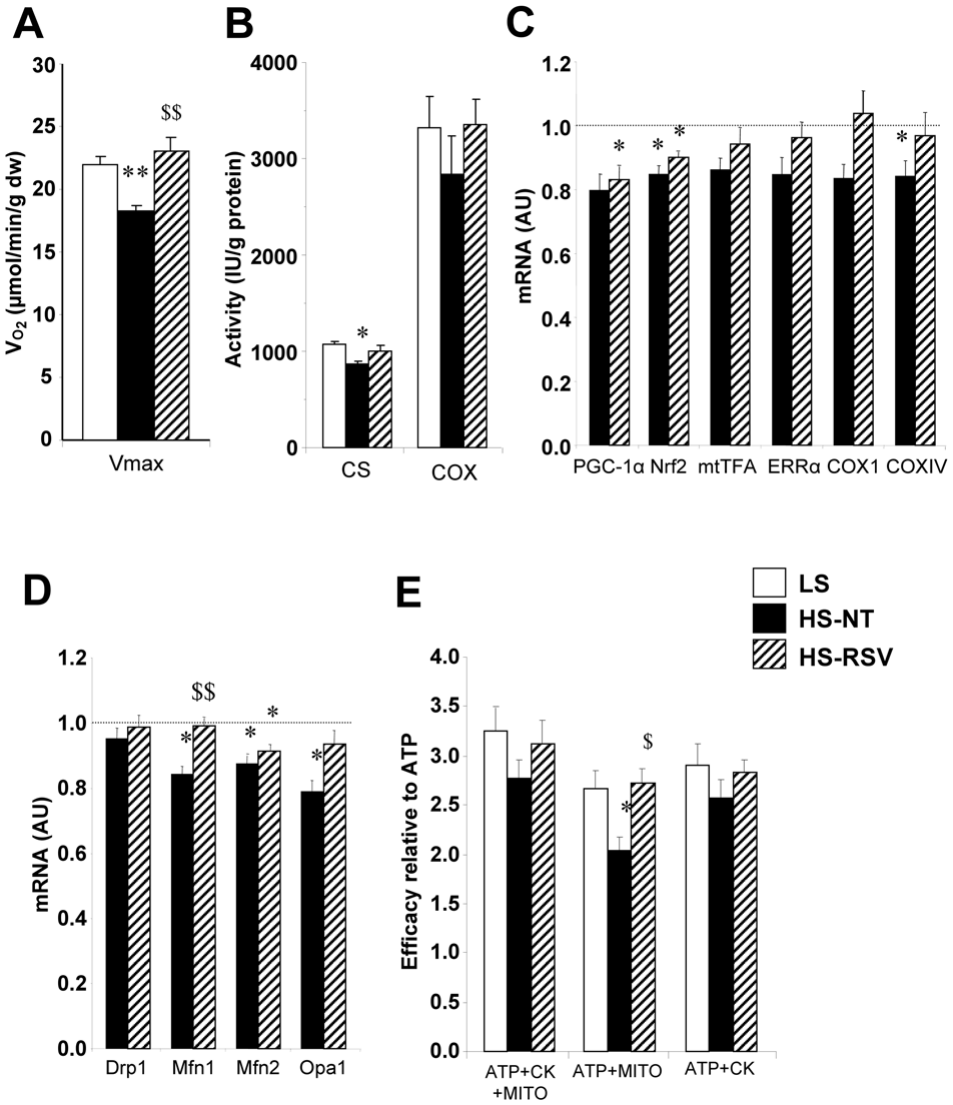
Cardiac mitochondrial function and biogenesis. (**A**) Maximal mitochondrial respiration of skinned fibers with 2 mM ADP, 4 mM malate, 0.1 mM palmitoylCoA, 0.15 mM palmitoylcarnitine, 1 mM pyruvate and 10 mM glutamate. (**B**) Citrate synthase (CS) and cytochrome oxidase (COX) activities, markers of mitochondrial mass. (**C**) mRNA levels of PGC-1α (peroxisome proliferator-activated receptor gamma coactivator 1α), Nrf2 (nuclear respiratory factor 2), mtTFA (mitochondrial transcription factor A), ERRα (estrogen-related receptor α), COX1 and IV (cytochrome c oxidase I and IV subunits). (**D**) mRNA levels of the fission protein Drp1 (dynamin-related protein 1) and the fusion proteins Mfn (mitofusin) 1 and 2 and OPA1 (optic atrophy 1). (**E**) Energetic coupling efficiency between sarcoplasmic reticulum ATPase and mitochondria or bound creatine kinases (CK) assessed by the ratio of calcium uptake under different energetic conditions (ATP+MITO+CK, ATP+MITO, ATP+CK, see Methods S1) to the uptake with ATP only. mRNA levels are normalized to LS group taken as 1. Between 6 and 9 animals per group. *P<0.05, **P<0.01, ***P<0.001 vs LS; ^$^P<0.05, ^$$^P<0.01 vs HS-NT.

Both maximal cardiac fiber oxidative capacity and citrate synthase (CS) activity were reduced in HS-NT rats but were preserved in HS-RSV rats compared with LS controls (Figure 4A and B), while COX activity was similar in all three groups. Expression levels of genes involved in energy metabolism are presented in Figure S1. There was a modest decrease in the expression of PGC-1α and its transcriptional cascade, key modulators of mitochondrial biogenesis, which were not prevented by resveratrol treatment (Figure 4C). Interestingly, expression of proteins involved in mitochondrial fusion was decreased in HS-NT animals, two of which (Mfn1 and OPA1) were restored in HS-RSV rats (Figure 4D).

Sarcoplasmic reticulum (SR) calcium uptake capacity with all substrates was not changed (Table S3), whereas the ability of mitochondria to supply energy to the SR-ATPase was decreased in HS-NT versus LS animals and was completely preserved in HS-RSV animals (Figure 4E). No significant change was observed in energy supply by CK. This suggests that the close contacts between SR and mitochondria, necessary for efficient energy transfer, are preserved by resveratrol.

**Figure 5:**
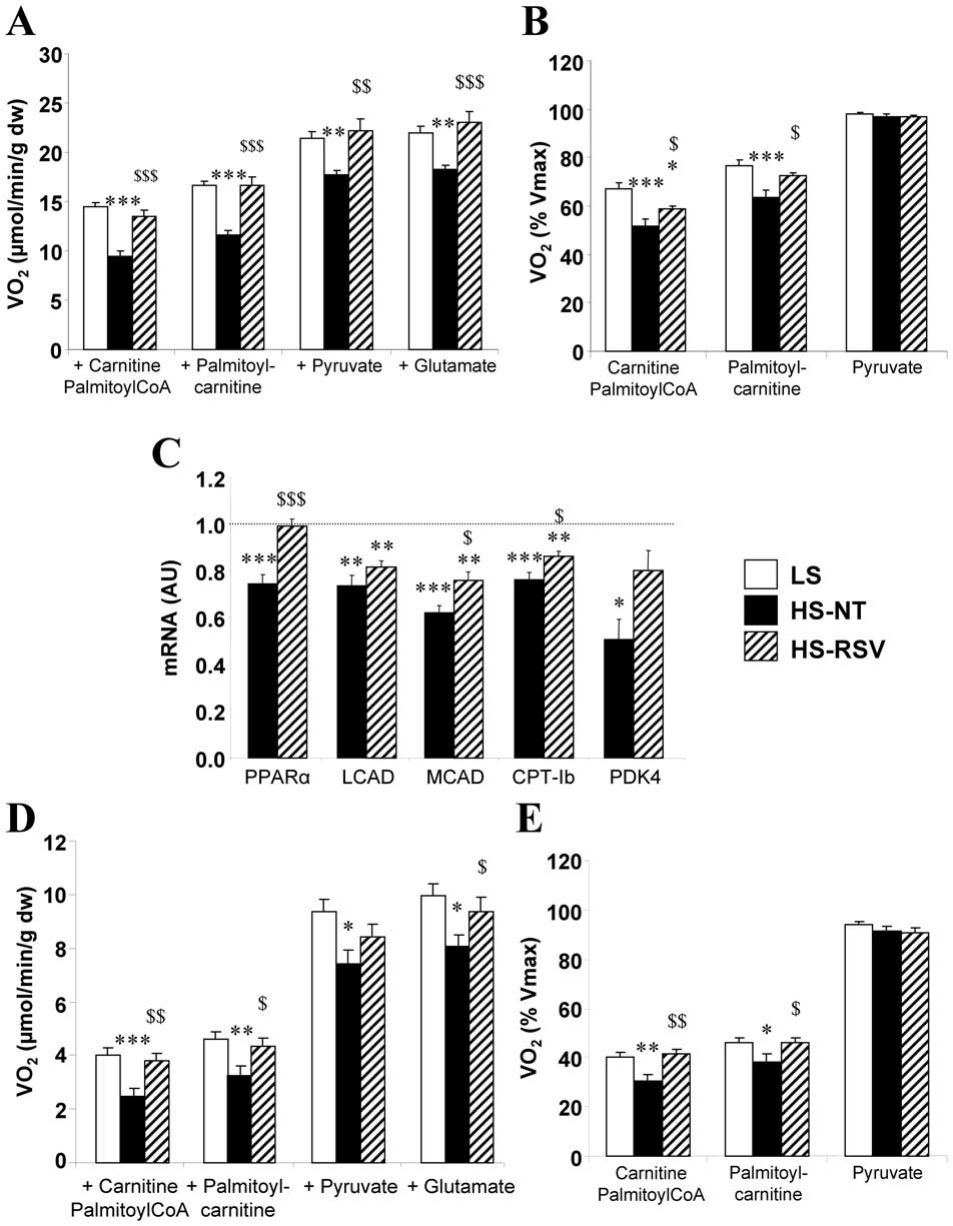
Resveratrol improves cardiac and soleus muscle fatty acid utilization. (**A**) Mitochondrial substrate utilization assessed by respiration of permeabilized cardiac fibers using cumulative addition of 2 mM ADP 4 mM malate, 1 mM carnitine and 0.1 mM palmitoylCoA, 0.15 mM palmitoylcarnitine, 1 mM pyruvate and 10 mM glutamate. (**B**) Respiration normalized to the maximal respiration rate and measured in the presence of all of the substrates revealed decreased fatty acid oxidation. (**C**) mRNA levels of PPARα (peroxisome proliferator-activated receptor α), LCAD (long-chain acylCoA dehydrogenase), MCAD (middle-chain acylCoA dehydrogenase), CPT-Ib (carnitine palmitoyltransferase Ib), PDK4 (pyruvate dehydrogenase kinase 4). (**D**) Mitochondrial substrate utilization of permeabilized soleus fibers. (**E**) Normalized respiration, indicating decreased fatty acid oxidation by soleus mitochondria. Between 7 and 9 animals per group. mRNA levels were normalized to LS group taken as 1. *P<0.05, **P<0.01, ***P<0.001 vs LS; ^$^P<0.05, ^$$^P<0.01, ^$$$^P<0.001 vs HS-NT.

Decreased FAO is an early event in the failing heart. Addition of palmitoyl CoA+carnitine, palmytoylcarnitine, pyruvate, and glutamate resulted in lower respiration rates in HS-NT compared to LS rats, and this was completely prevented with resveratrol treatment (Figure 5A). Moreover, when respiration was normalized to the maximal respiration rate in the presence of all substrates (Figure 5B), the results clearly showed a selective decrease in mitochondrial FAO in HS-NT rats, which was prevented with resveratrol treatment. Accordingly, expression of the nuclear transcription factor controlling FAO, PPARα, as well as its target genes involved in FA transport (CPT-1b) and oxidation (MCAD, LCAD), or carbohydrate utilization (PDK4), was decreased in the heart of HS-NT. Expression level of PPARα, CPT-1b and MCAD was preserved in HS-RSV animals compared with LS animals (Figure 5C).

Skeletal muscles also suffer from energy starvation in HF. In soleus muscle as in cardiac muscle, the decrease in oxidative capacity was preserved by resveratrol (Figure 5D) as was the specific decrease in mitochondrial fatty acid oxidation (Figure 5E).

### Signaling pathways potentially involved in beneficial effects of resveratrol

**Figure 6:**
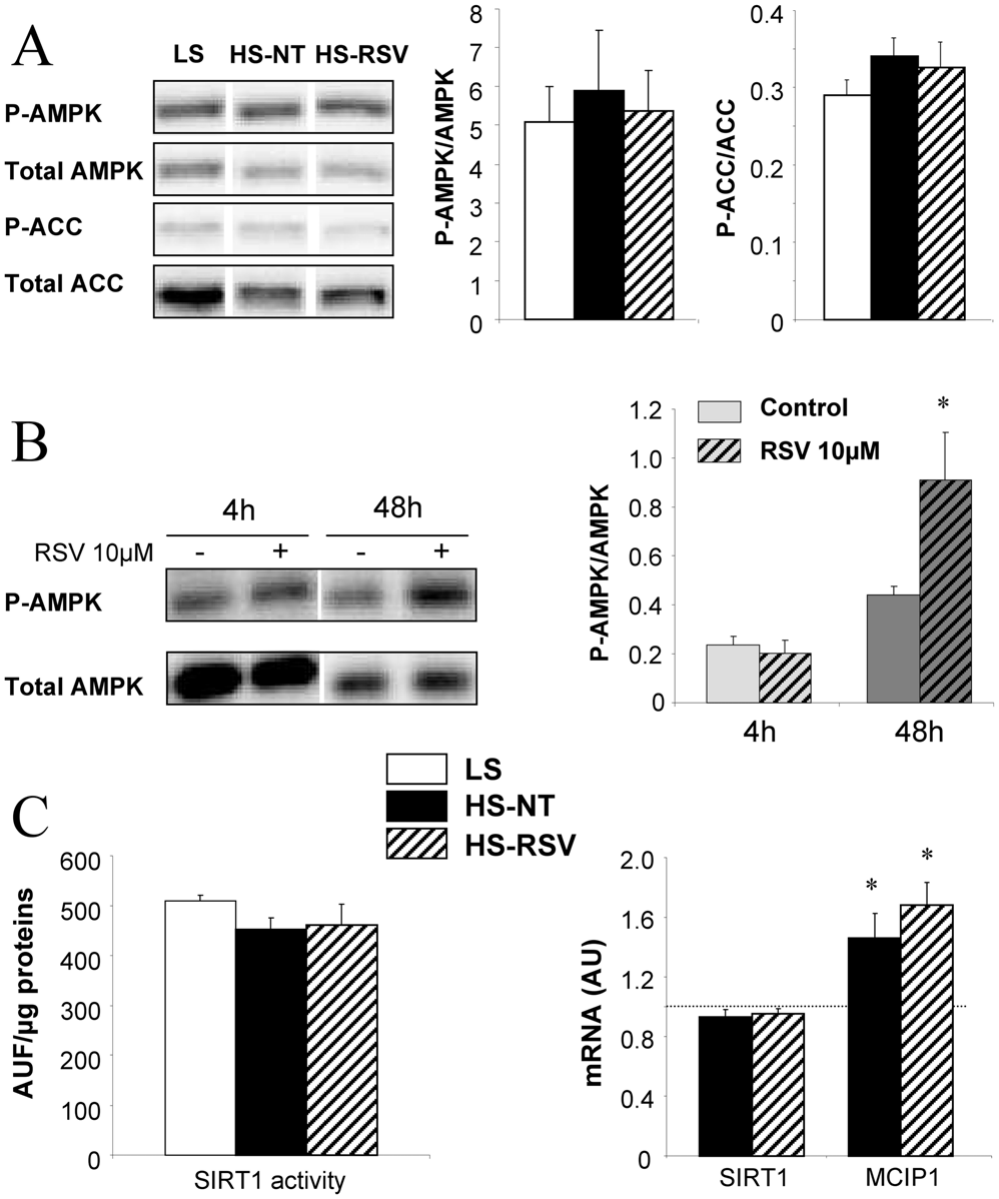
Resveratrol and signaling pathways. (**A**) Total and phosphorylated AMPK (AMP-activated kinase) and ACC (acylCoA carboxylase) were unchanged in ventricles from LS (n = 7), HS-NT (n = 6) and HS-RSV (n = 7) groups. (**B**) AMPK activation induced in adult rat ventricular myocytes after 48 h of resveratrol treatment (n = 6). (**C**) SIRT1 activity and expression were unchanged, and calcineurin transcriptional activity assessed by measuring the expression of its target gene MCIP1 was increased in HS-NT rats but not affected by resveratrol treatment. mRNA levels normalized to LS group taken as 1. *P<0.05, **P<0.01 vs LS or control.

AMPK protein content and phosphorylation as well as phosphorylation of ACC, its main target, did not differ between groups at the end of experiments (Figure 6A). However AMPK activity showed an early increase after 48 hours in RSV-treated cardiomyocytes (Figure 6B). Calcineurin transcriptional activity was estimated by measuring the expression of MCIP1, a known target of NFAT [25]. MCIP1 expression was increased in HS-NT and not influenced by resveratrol (Figure 6C), in accordance with the maintained hypertrophy. Neither expression nor activity of SIRT1 was changed (Figure 6C).

## Discussion

The main purpose of this study was to investigate the use of resveratrol as a potential metabolic therapy for hypertension-induced HF. Resveratrol treatment resulted in 1) an increase in survival rate by more than 50%; 2) complete preservation of myocardial function; 3) prevention of endothelial dysfunction; and 4) preservation of cardiac and skeletal muscle mitochondrial respiration, energy supply and FA utilization, a well as cardiac energy transfer. These beneficial effects of resveratrol occurred despite the continued presence of cardiac hypertrophy and hypertension in this model.

### DSS rats present signs of heart failure

The high salt diet rapidly induced severe hypertension followed by ventricular hypertrophy and LV dysfunction without dilatation, hypercholesterolemia and a high mortality rate. In parallel, markers of cardiac remodeling were sharply upregulated; metabolic markers of HF such as CK isoenzymes and CS were decreased while calcium metabolism markers were not significantly altered. However, there was no clear sign of pulmonary edema and no ascites. Severe body wasting, which is associated with poor prognosis in HF [26], started at five weeks and was associated with increased mortality rate. DSS rats thus developed ventricular dysfunction with early signs of HF.

### Resveratrol treatment improves survival and cardiac function

Treatment of HS rats with resveratrol improved cardiac function, concomitant with a remarkable increase in survival rate. The high mortality rate of DSS rats is due *inter alia* to stroke, renal failure and HF. As already reported [27], resveratrol blunted the up-regulation of stress markers, suggesting attenuation of the stress response of the myocardium. These results match those obtained in SHR and infarcted rats where cardiac function was preserved by resveratrol without affecting BP [17], [18], [27]. In DSS rats, the renal hypertrophy is mainly the result of fibrosis induced by activated inflammatory or immune processes, which, in turn, depend on oxidative stress [28], while aldosterone seems to play a role on glomerulosclerosis, podocyte [29] non-related to the hypertension. This effect could result from local anti-oxidant activity of RSV.

Interestingly, in line with the maintained elevated BP, improvement of cardiac function and survival in DSS rats was not accompanied by a prevention of ventricular wall thickening and hypertrophy. The anti-hypertrophic effects of resveratrol was reported in animal models [30] associated with the circulating renin-angiotensin system (RAS) activation. This system may be blunted by RSV treatment [22], [31] thus reducing the cardiac hypertrophy. In contrast, in DSS rats, circulating RAS is rather inhibited [32] and hypertension is mostly linked to blood volume expansion [33] so that potential anti-RAS activity of RSV does not reduce hypertension. This suggests that resveratrol may have separate, beneficial effects on hypertension and cardiac hypertrophy, and thus the benefits of resveratrol may depend on the etiology of HF. Interestingly, stimulation of carbohydrate metabolism by activation of pyruvate kinase induced similar beneficial effects without reducing BP and hypertrophy [34].

### Resveratrol prevents endothelial dysfunction

Another pathological event in HF is endothelial dysfunction resulting in increased peripheral resistances and increased load to the myocardium. This endothelial dysfunction is due to a decrease in NO production resulting in impaired smooth muscle relaxation. In HS-NT animals, the impaired vasodilatation could be attributed to the sharp decrease in eNOS protein content inducing a decrease in NO production. The moderate decrease in the sensitivity of vascular relaxation to NO may also play a role in the reduced response to carbachol.

DSS rats also demonstrate high level of vascular fibrosis found in aortic tunica media and tunica adventitia [35], which increases the vascular rigidity thus contributing to the hypertension. Probably, such rigidity was not corrected by RSV and this explains why improved endothelial function in aorta did not lead to an attenuation of the hypertension. Further studies on small arteries need to be conducted in order to elucidate this question.

Polyphenols are known to exert positive effects on endothelial function and cardiac function in obesity [36]. The remarkable improvement of endothelial function by resveratrol in HS animals treated with RSV could be achieved through up-regulation of eNOS expression that normalizes vasodilatation.

### Resveratrol improves mitochondrial function, fatty acid utilization and energy transfer

Altered energy metabolism, particularly mitochondrial dysfunction, is hallmark of HF that affects both cardiac and skeletal muscles [5], [6], [37], [38]. HS-NT rats presented signs of myocardial metabolic disorders with reduced oxidative capacity, CS activity and miCK content, and a global downregulation of genes involved in mitochondrial biogenesis such as PGC-1α, ERRα and their downstream target genes, similar to numerous HF models including DSS rats [8]–[10], [12], [34], [34], [39,40]. Resveratrol preserved cardiac and skeletal muscle oxidative capacity and mitochondrial enzyme activities, with no major changes in expression of the mitochondrial biogenesis transcription cascade.

In HS-NT rats, the specific reduction in mitochondrial FAO capacity was accompanied by down-regulation of genes controlling FAO, PPARα and its downstream targets. Remarkably, these alterations were prevented by resveratrol treatment, independently of changes in triglyceridemia. These results are consistent with the beneficial effects of grape extracts on PPARα and PGC-1α observed in DSS rats [23] and the PPARα-dependent beneficial effect of resveratrol on isoprenaline-induced cardiac dysfunction [41]. Interestingly, our results indicate that FAO activation can be associated with improved cardiac function, provided that a concomitant increase in mitochondrial capacity ensures proper FA utilization.

Decreased energy transfer by the CK system is also a hallmark of HF [37], characterized by a decrease in total and miCK activity, the latter being completely normalized by resveratrol treatment. This suggests that maintaining energy metabolism can help preserve cardiac contractile function and survival.

Mitochondria are involved in permanent fusion and fission events. Fission requires the dynamin-related protein 1 (Drp1), whereas fusion is mediated by two dynamin-related proteins involved in outer membrane fusion, mitofusins (Mfn1 and Mfn2), and OPA1 (Optic atrophic type 1 protein) which is localized at the inner membrane (for review see [42]). In HS-NT rats, expression of fusion proteins was decreased, suggesting a shift towards increased fragmentation of the mitochondrial network. Again, resveratrol treatment prevented Mfn1 and OPA1 changes thus tending to normalize the fusion/fission balance.

Finally, resveratrol beneficial effects on mitochondrial biogenesis and dynamics may explain the conservation of energetic coupling between mitochondria and the SR-ATPase. Direct energy cross talk between mitochondria and energy consuming sites [43] is driven by restricted diffusion of adenine nucleotides and is facilitated by the juxtaposition of sites that produce and consume energy. HF is characterized by impaired intracellular organization and a decrease in SR calcium storage capacity, which is directly linked to a local decrease in ATP/ADP ratio [44]. Such a defect in mitochondria/SR-ATPase interaction was also evident in the HS-NT group, and was again prevented by resveratrol treatment. This reveals a novel effect of resveratrol on energy transfer and supply.

Importantly, these resveratrol-mediated effects are not a consequence of reduced hypertension or cardiac hypertrophy.

### Mode of action of resveratrol

**Figure 7:**
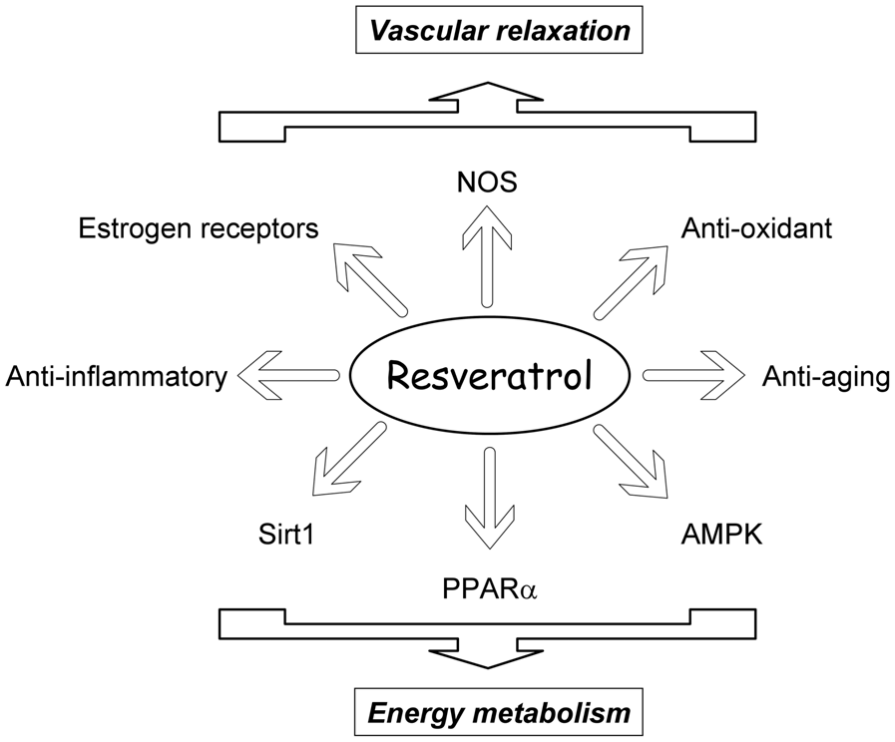
Summary of possible modes of action of resveratrol.

In view of the pleiotropic effects of RSV, it is likely that the beneficial effects of resveratrol in HF are multifactorial and involve multiple signaling pathways acting on a variety of cellular targets (Figure 7). The major effect of resveratrol was to abolish the down-regulation of PPARα and modestly prevent PGC-1α transcription cascade and mitochondrial lipid oxidation decrease. Interestingly, the cardioprotective and neuroprotective effects of resveratrol (and grapes) were shown to depend on PPARα and to involve improved lipid metabolism and inflammation [23], [41], [45]. Resveratrol effects could in part be mediated by activation of PGC-1α via SIRT1-dependent deacetylation as seen in skeletal muscle [46] but cardiac SIRT1 expression and activity were not altered in HS-NT and HS-RSV groups. However, lack of effect of resveratrol on PGC-1α acetylation in heart, was already reported in resveratrol-treated mice [14]. AMPK, is a central target for the metabolic effects of resveratrol and an important player in modulating energy metabolism [15]. Although activated for short term resveratrol treatment of cardiomyocytes, AMPK activation was unchanged in HS-NT and resveratrol-treated rats at the end of treatment. The increased calcineurin transcriptional activity in HS-NT rats was not prevented by resveratrol, consistent with the maintained hypertrophy. Moreover it should be noted that resveratrol may also indirectly improve cardiac energy metabolism through its vasorelaxing effect, which could ameliorate perfusion and enhance oxygen and substrate delivery to the heart and the periphery. Finally, the beneficial cardiovascular and metabolic effects of resveratrol could also result from its phytooestrogenic properties [47], [48].

### Conclusions

The present study was aimed at investigating the relevance of a metabolic therapy of HF using resveratrol. Our data show multiple beneficial effects of resveratrol in hypertension-induced HF, with increased survival, reduced body wasting and improved cardiac and endothelial function in particular. The major metabolic effect of resveratrol in both cardiac and skeletal muscles was to restore the expression of PPARα and attenuate PGC-1α transcription cascade down-regulation and mitochondrial lipid oxidation decrease. Interestingly, the cardioprotective and neuroprotective effects of resveratrol (and grapes) were shown to depend on PPARα and to involve improved lipid metabolism and inflammation [23], [41], [45]. Indeed, resveratrol completely prevented cardiac FA utilization reduction and improved mitochondria-driven energy supply while preserving expression of mitochondrial fusion protein. This suggests that amelioration of energy metabolism may contribute to the regression of HF independent of cardiac hypertrophy. Interestingly, a recent study describing the metabolic remodeling of the failing heart showed that activation of carbohydrate metabolism with dichloroacetic acid provided a similar improvement in cardiac function and survival [34]. Altogether this highlights the relevance of metabolic therapy in HF. Resveratrol and other metabolic activators are thus interesting candidates for preventive metabolic therapy in HF.

## Methods

Detailed Methods are presented in the supplemental file (Methods S1).

### Ethics Statements

All experiments were performed in conformity with the European Community guiding principles in the care and use of animals (Directive 2010/63/EU of the European Parliament). Authorizations to conduct animal experiments were obtained from the French Ministère de l'Agriculture, de la Pêche et de l'Alimentation (no. 92-284, June 27, 2007).

### Animals conditioning

The Dahl salt-sensitive rats (DSS) were chosen as a model of hypertension. Seven week-old male DSS rats (Charles River France) were randomly divided in two groups: 9 rats were fed a low-salt diet (0.3% NaCl) for 11 weeks as controls (LS rats) and 62 rats were fed a high-salt diet (8% NaCl) for 3 weeks (HS rats). The HS group was then subdivided into two groups for 8 weeks: a non-treated group (HS-NT; n = 48) and a resveratrol-treated group (HS-RSV; n = 14; 18 mg/kg/day in the diet). Diets were manufactured by UPAE-INRA (INRA, Jouy-en-Josas, France) and composition is given in Table S4. The animals were housed 1 per cage in a temperature-controlled room (22°C), with a 12/12 h light/dark cycle, and were provided food and water ad libitum.

Diets were manufactured by UPAE-INRA (INRA, Jouy-en-Josas, France) and composition is given in Table S1. The dose of resveratrol of ≈20 mg/kg/d proved to be efficient in preventing pulmonary hypertension [49] and in vasoprotection [50] and to have no toxicity [51].

### Echocardiography and blood pressure measurements

Echocardiography was performed at 3 and 11 weeks of diet treatment using a 12 MHz transducer (Vivid 7, General Electric Healthcare) under 2.5% isoflurane gas anesthesia. Two-dimensional-guided (2D) M-mode echocardiography was used to determine wall thickness and left ventricular chamber diameter at systole and diastole, and contractile parameters such as fractional shortening (FS) or ejection fraction (EF%). Left ventricular mass was calculated according to the Penn formula home-adapted for the rat heart: (LVM = 1.04×[(Dtd+SIV+PP)^3^−(Dtd)^3^]). Doppler at the mitral valve was used to measure E and A waves when possible and Doppler tissue imaging was used to assess early diastolic lateral mitral annulus velocity as an estimate of left ventricle diastolic function [24].

Arterial blood pressure was measured in conscious, restrained rats using a tail-cuff system (CODA™ 2 system, EMKA Technologies France) in a dark temperature-controlled room (22°C) in the morning. Rats were subjected to 15 acclimation measurements in restraint holder then blood pressure was calculated from the average of 10 measurement cycles.

### Blood assays and sacrifices

At the end of the conditioning period, all non-fasted rats were anesthetized by intraperitoneal injection of pentobarbital (200 mg/kg) at 10 am. Depth of anesthesia was checked by toe pinch before the start of surgery. Whole blood was sampled for lipid and glucose profile analysis using a clinical Cholestech LDX blood analyzer (Cholestech LDX system, GaiaMed). The heart was then quickly removed for euthanasia then soleus muscles and aorta were collected. Part of tissue was immediately used for functional experiments, and another part was rapidly frozen and kept at −80°C for further investigations.

### Aortic reactivity

Vascular function was studied on aorta rings with preserved endothelium mounted in a myograph under optimal and stabilized resting tension. After a 60 min equilibration period in Krebs solution [52], contractile capacity of arteries was evaluated using Krebs solution containing 80 mM of KCl. A submaximal concentration of prostaglandin F2α (PGF2α) was applied to produce a pre-contraction of 80%. Endothelium-dependent relaxation was then assessed by the addition of increasing amounts of carbachol (10^−9^ to 3×10^−5^ M) while endothelium-independent relaxation was assessed directly using the nitric oxide (NO) donor S-nitrosothiol-N-acetyl-penicillamine (SNAP, 10^−9^ to 10^−5^ M).

### Mitochondrial function

Oxygen consumption was measured on saponin-permeabilized cardiac and soleus fibers [53]. Maximal respiration rates and sensitivity of mitochondrial respiration to carbohydrates and fatty acids were evaluated using an adapted protocol [54]. Oxygen consumption was followed in the presence of 2 mM ADP and 4 mM malate, by successive additions of substrates: palmitoylCoA 0.1 mM plus carnitine 1 mM, palmitoylcarnitine 0.15 mM, pyruvate 1 mM and glutamate 10 mM. Maximal respiration was measured in the presence of all these mitochondrial substrates. Rates of respiration are given in µmoles O_2/_min/g dry weight (dw). Two to three experiments were performed for each muscle and each protocol.

### Energy supply on SERCA activity

Local energy control of the sarcoplasmic reticulum (SR) ATPase was assessed using saponin-skinned fibers from papillary muscle mounted on a stainless-steel hook with a force transducer (AE 801, Microelectronics, Horton, Norway) as previously described [43]. Fibers were immersed in 2.5 ml chambers placed into a temperature-controlled bath at 22°C. Briefly, SR calcium loading was initiated at pCa 6.5 in the presence of exogenous ATP, with or without endogenous mitochondrial ATP production and/or creatine kinase system activation. Azide (2 mM) was used to block mitochondrial ATP production, and solutions lacking phosphocreatine (PCr used at 12 mM) were used to block creatine kinase activity. SR calcium release was elicited with 5 mM caffeine, and was detected by measuring the resulting contractile force transient. At the end of each experiment pCa–tension relationship was assessed by stepwise increasing the calcium concentration in the presence of caffeine and was used as an endogenous calibration of calcium concentration around the fibers. [Calcium]-time integral was calculated to estimate calcium uptake occurring before caffeine challenge. Results are expressed as absolute value for MITO+PCr or relative to the load with ATP alone. pCa-tension relationship allowed to calculate resting and maximal developed force. Force was normalized to cross sectional fiber area and expressed as mN.mm^−2^. Data were analyzed using a linearization of the Hill equation F (relative force) = [Ca^nH^/(K+[Ca]). The Hill coefficient (nH) and the pCa for half maximal activation pCa_50_ = (−log_10_ K)/^nH^ were computed.

### Real-time quantitative PCR analysis

Real-time PCR was performed using TaqMan Low Density Array (TLDA) technology. TLDA were designed to amplify 48 cDNA for each sample as follows: 43 target genes involved in energy metabolism and mitochondrial function and 5 housekeeping genes (Table S5). For more details see Methods S1 available in the online supporting information.

### Immunoblotting

SERCA2, calsequestrin and eNOS expression, and AMPK phosphorylation were assessed on proteins extracts from hearts or aorta and/or resveratrol-treated adult rat ventricular myocytes by immunobloting as detailed in Methods S1.

### Biochemical studies

Frozen tissue samples were weighed, homogenized (Bertin Precellys 24) in ice-cold buffer (50 mg/ml) containing (mM) 5 HEPES (pH 8.7), 1 EGTA, 1 DTT and 0.1% Triton X-100. Citrate synthase (CS), cytochrome oxidase (COX) and total creatine kinase (CK) as well as CK isoforms activities were determined in homogenized ventricles as previously described [37].

SIRT1 activity was assessed in ventricles using a SIRT1 fluorimetric assay kit (BIOMOL, EnzoLifeSciences) following an adapted procedure. Briefly, 25 µg of total protein were incubated with 15 µl of Fluor de Lys-Sirt1 substrate (100 µM) and NAD^+^ (100 µM) at 37°C for 30 min. The reaction was stopped by the addition of 50 µl of developer reagent containing nicotinamide (2 mM) and the fluorescence was subsequently monitored for 45 min at 360 nm (excitation) and 460 nm (emission). The change in fluorescence (arbitrary fluorescence units, AFU) per minute was normalized to the amount of total protein in the sample.

### Statistical analysis

Data are expressed as mean±SEM. The survival of the animals was analyzed by the Kaplan-Meier method with a log-rank test. Other data were analyzed by ANOVA followed by Student–Newman–Keuls post hoc test. For aortic relaxation, curves have been analyzed using repeated measure ANOVA followed by Tukey's multiple comparison test. Statistical significance between groups was defined as *p*<0.05.

## Supporting Information

Figure S1 **mRNA expression of mitochondrial and energy metabolism selected proteins.** This figure includes total results of TLDA experiments. *P<0.05 vs LS; ^$^P<0.05 vs HS-NT.
(TIF)

Table S1 **Lipid and glucose profile in blood samples.** Total cholesterol, HDL and LDL were significantly increased in HS-NT compared to LS animals and tended to decrease with RSV treatment. No significant change in blood triglycerides and glucose was observed whatever the groups.
(DOC)

Table S2 **Echocardiography and blood pressure measurements.** Table shows all echocardiographic analysis and blood pressure data performed at 3 weeks and 11 weeks. As HS-NT and HS-RSV groups showed no difference at 3 weeks, data were pooled (HS group). At 11 weeks, systolic and diastolic dysfunction was evident in HS-NT animals and could be largely prevented by resveratrol treatment. Resting, maximal and mean blood pressures were increased in HS-NT animals but were not improved by resveratrol treatment.
(DOC)

Table S3 **Mechanical parameters and calcium homeostasis.** No major change in force and calcium sensitivity was observed in HS-NT rats except a decrease in the Hill coefficient that was not prevented by resveratrol. The tendency to decrease of the maximal SR calcium content (with optimal substrate ATP+MITO+PCr) and of SERCA2 protein content in HS-NT rats was prevented by resveratrol but these results did not reach significance.
(DOC)

Table S4 **Composition of the diet.**
(DOC)

Table S5 **Design of TaqMan Low Density Arrays.**
(DOC)

Methods S1 **Supplementary Methods and Results.**
(DOC)
