# Supplementary figures and images for: Resveratrol Improves Survival, Hemodynamics and Energetics in a Rat Model of Hypertension Leading to Heart Failure
Source: PLoS One. 2011 Oct 18;6(10):e26391. doi: 10.1371/journal.pone.0026391 (PMC3196575; doi:10.1371/journal.pone.0026391)

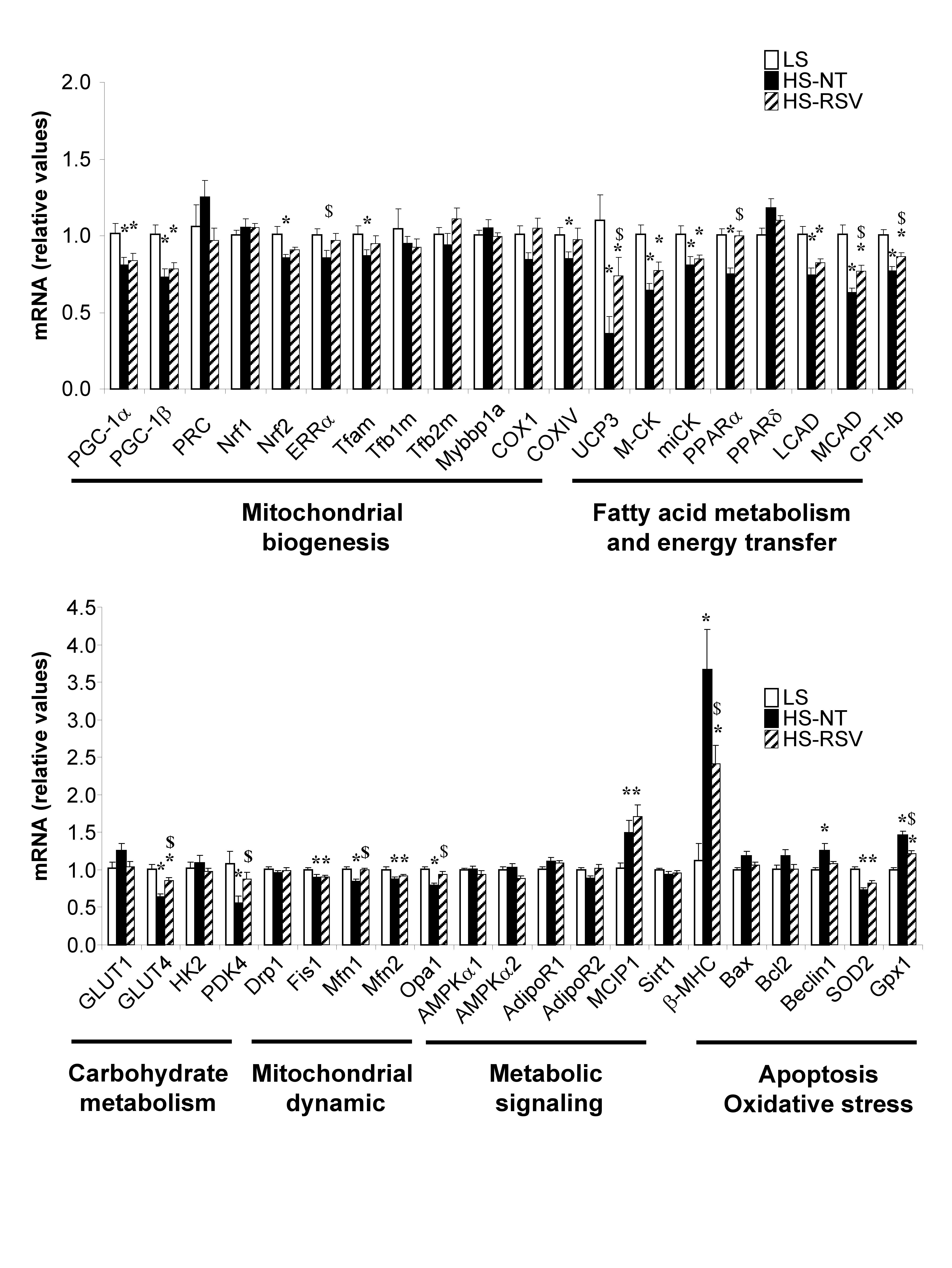

Supplement: Figure S1 — mRNA expression of mitochondrial and energy metabolism selected proteins. This figure includes total results of TLDA experiments. *P<0.05 vs LS; $P<0.05 vs HS-NT. (TIF) [file pone.0026391.s001.tif]
