# Supplementary material for: Resveratrol Improves Survival, Hemodynamics and Energetics in a Rat Model of Hypertension Leading to Heart Failure
Source: PLoS One. 2011 Oct 18;6(10):e26391. doi: 10.1371/journal.pone.0026391 (PMC3196575; doi:10.1371/journal.pone.0026391)
Supplement: Table S1 — Lipid and glucose profile in blood samples. Total cholesterol, HDL and LDL were significantly increased in HS-NT compared to LS animals and tended to decrease with RSV treatment. No significant change in blood triglycerides and glucose was observed whatever the groups. (DOC) [file pone.0026391.s002.doc]

**Table S1. Lipid and glucose profile in blood samples.**

|  | **LS** | **HS-NT** | **HS-RSV** |
| --- | --- | --- | --- |
| **Total cholesterol** | 105±4 | 206±30*** | 167±15** |
| **HDL cholesterol** | 36±3 | 60±14* | 42±4 |
| **LDL cholesterol** | 28±4 | 115±31** | 86±13* |
| **Triglyceridemia** | 203±15 | 160±34 | 196±26 |
| **Glycemia** | 189±9 | 157±7 | 163±9 |

Data are expressed in mg/dl. *P<0.05, **P<0.01, ***P<0.001 vs LS.
