# Supplementary material for: Resveratrol Improves Survival, Hemodynamics and Energetics in a Rat Model of Hypertension Leading to Heart Failure
Source: PLoS One. 2011 Oct 18;6(10):e26391. doi: 10.1371/journal.pone.0026391 (PMC3196575; doi:10.1371/journal.pone.0026391)
Supplement: Table S2 — Echocardiography and blood pressure measurements. Table shows all echocardiographic analysis and blood pressure data performed at 3 weeks and 11 weeks. As HS-NT and HS-RSV groups showed no difference at 3 weeks, data were pooled (HS group). At 11 weeks, systolic and diastolic dysfunction was evident in HS-NT animals and could be largely prevented by resveratrol treatment. Resting, maximal and mean blood pressures were increased in HS-NT animals but were not improved by resveratrol treatment. (DOC) [file pone.0026391.s003.doc]

**Table S2. Echocardiography and blood pressure measurements.**

|  | **3 weeks** | | **11 weeks** | | |
| --- | --- | --- | --- | --- | --- |
|  | **LS**  **N=8** | **HS-NT**  **N=18** | **LS**  **N=8** | **HS-NT**  **N=8** | **HS-RSV**  **N=9** |
| **Blood pressure** |  |  |  |  |  |
| Diastolic blood pressure | 98±9 | 133±4 | 97±7 | 116±11* | 131±9* |
| Systolic blood pressure | 155±9 | 191±4 | 147±6 | 190±5*** | 197±5*** |
| Mean blood pressure | 116±9 | 152±4 | 113±6 | 140±9** | 153±7** |
| **Echocardiography** |  |  |  |  |  |
| Heart rate (beats/min) | 395±5 | 363±7* | 374±6 | 290±20*** | 357±11^$$$^ |
| IVSd (mm) | 1.51±0.08 | 1.82±0.07* | 1.61±0.10 | 2.06±0.12* | 1.93±0.07* |
| IVSs (mm) | 2.93±0.12 | 3.34±0.11* | 3.09±0.18 | 3.12±0.21 | 3.25±0.08 |
| LVIDd (mm) | 7.37±0.09 | 7.21±0.15 | 7.4±0.13 | 8.18±0.38 | 7.6±0.21 |
| LVIDs (mm) | 3.27±0.11 | 2.84±0.18 | 3.03±0.14 | 5.03±0.38*** | 3.56±0.31^$$^ |
| LVPWd (mm) | 1.92±0.08 | 2.04±0.09 | 1.84±0.09 | 1.96±0.15 | 2.12±0.09 |
| LVPWs (mm) | 3.24±0.10 | 3.61±0.11* | 3.19±0.06 | 2.92±0.2 | 3.37±0.1^$^ |
| LVTDV (ml) | 0.90±0.03 | 0.86±0.05 | 0.91±0.04 | 1.23±0.16 | 0.99±0.08 |
| LVTSV (ml) | 0.09±0.01 | 0.07±0.01 | 0.08±0.01 | 0.34±0.06*** | 0.14±0.04^$$^ |
| EF (%) | 90±1 | 92±1 | 91.7±0.81 | 73.7±3.3*** | 87.4±2.25^$$$^ |
| Stroke volume (ml) | 0.81±0.03 | 0.79±0.04 | 0.83±0.04 | 0.89±0.12 | 0.85±0.05 |
| FS (%) | 56±1 | 61±2 | 59.2±1.4 | 38.9±2.8*** | 53.7±2.9^$$$^ |
| LV mass (mg) | 896±43 | 1020±38* | 913±50 | 1339±119* | 1211±110* |
| Cardiac output (ml/min) | 318±11 | 283±14 | 311±13 | 251±24* | 300±12^$^ |
| **Doppler** |  |  | **N=5** | **N=6** | **N=9** |
| E (m/s) | nd | nd | 1.10±0.03 | 0.99±0.13 | 1.12±0.06 |
| Eann (m/s) | nd | nd | 0.082±0.010 | 0.046±0.003 *** | 0.067±0.003***^,$$^ |
| E/Eann ratio | nd | nd | 14.2±1.9 | 22.0±3.2* | 17.1±1.2 |
| IVRT (msec) | nd | nd | 26.8±2.4 | 30.8±2.8 | 27.1±1.2 |

Data are expressed as means ± SEM. IVSd, interventricular septal thickness at end-diastole; IVSs, interventricular septal thickness at end-systole; LVIDd, left ventricular internal dimension at end-diastole; LVIDs, left ventricular internal dimension at end-systole; LVPWd, left ventricular posterior wall thickness at end-diastole; LVPWs, left ventricular posterior wall thickness at end-systole; LVTDV, left ventricular telediastolic volume, LVTSV, left ventricular telesystolic volume; EF, ejection fraction; FS, fractional shortening; E, E wave amplitude, Eann, early diastolic lateral mitral annulus velocity; IVRT, isovolumic relaxation time. N= number of animals.

nd, not determined. *P<0.05, ***P<0.001 vs LS; ^$^P<0.05, ^$$^P<0.01, ^$$$^P<0.001 vs HS-NT.
