# Supplementary material for: Resveratrol Improves Survival, Hemodynamics and Energetics in a Rat Model of Hypertension Leading to Heart Failure
Source: PLoS One. 2011 Oct 18;6(10):e26391. doi: 10.1371/journal.pone.0026391 (PMC3196575; doi:10.1371/journal.pone.0026391)
Supplement: Table S3 — Mechanical parameters and calcium homeostasis. No major change in force and calcium sensitivity was observed in HS-NT rats except a decrease in the Hill coefficient that was not prevented by resveratrol. The tendency to decrease of the maximal SR calcium content (with optimal substrate ATP+MITO+PCr) and of SERCA2 protein content in HS-NT rats was prevented by resveratrol but these results did not reach significance. (DOC) [file pone.0026391.s004.doc]

**Table S3.** Mechanical parameters and calcium homeostasis

|  | **LS**  **N=6, n=19** | **HS-NT**  **N=7, n=20** | **HS-RSV**  **N=7, =20** |
| --- | --- | --- | --- |
| **Myofilament properties** |  |  |  |
| Resting Force (mN.mm^-2^) | 5.03±0.43 | 4.38±0.30 | 4.29±0.34 |
| Maximal Force (mN.mm^-2^) | 25.3±1.1 | 23.2±0.7 | 24.8±0.8 |
| pCa_50_ | 5.87±0.01 | 5.86±0.02 | 5.88±0.01 |
| Hill coeffficient | 4.95±0.33 | 3.84±0.22* | 3.98±0.31* |
| **Sarcoplasmic reticulum** |  |  |  |
| Surface calcium (µM.sec) | 52.1±1.4 | 47.2±1.7 | 49.3±2.2 |
| SERCA_2_ protein | 13.9±2.9 | 9.2±0.7 | 13.0±1.1 |
| Calsequestrin protein | 4.7±0.3 | 4.8±0.3 | 5.7±0.9 |

Data are expressed as means ± SEM. N= number of animals; n= number of fibers. *P<0.05.
