# Supplementary material for: Resveratrol Improves Survival, Hemodynamics and Energetics in a Rat Model of Hypertension Leading to Heart Failure
Source: PLoS One. 2011 Oct 18;6(10):e26391. doi: 10.1371/journal.pone.0026391 (PMC3196575; doi:10.1371/journal.pone.0026391)
Supplement: Table S4 — Composition of the diet. (DOC) [file pone.0026391.s005.doc]

**Table S4. Composition of the diet in g/kg**

| **Ingredients** | **LS diet** | **HS diet** | **HS+RSV diet** |
| --- | --- | --- | --- |
| **Casein** | 200 | 200 | 200 |
| **Dl-methionine** | 3 | 3 | 3 |
| **Corn starch** | 450 | 450 | 450 |
| **Saccharose** | 200 | 200 | 200 |
| **Cellulose** | 50 | 50 | 50 |
| **Corn oil** | 50 | 50 | 50 |
| **Mineral mix (AIN 93)** | 35 | 35 | 35 |
| **Vitamin mix (AIN 93)** | 10 | 10 | 10 |
| **Choline hydrochloride** | 2 | 2 | 2 |
| **Extra NaCl** |  | 80 | 80 |
| **Resveratrol** |  |  | 0.35 (0.035%) |
