# Supplementary material for: Resveratrol Improves Survival, Hemodynamics and Energetics in a Rat Model of Hypertension Leading to Heart Failure
Source: PLoS One. 2011 Oct 18;6(10):e26391. doi: 10.1371/journal.pone.0026391 (PMC3196575; doi:10.1371/journal.pone.0026391)
Supplement: Table S5 — Design of TaqMan Low Density Arrays. (DOC) [file pone.0026391.s006.doc]

**Table S5. Design of TaqMan Low Density Arrays**

| **Genes** | **Alias** | **Assay ID (Applied Biosystems)** |
| --- | --- | --- |
| **Acadl** | **LCAD** | Rn00563121_m1 |
| **Acadm** | **MCAD** | Rn00566390_m1 |
| **Adipoq** | **Adiponectine** | Rn00595250_m1 |
| **Adipor1** |  | Rn01483784_m1 |
| **Adipor2** |  | Rn01463173_m1 |
| **Bax** |  | Rn02532082_g1 |
| **Bcl2** |  | Rn99999125_m1 |
| **Becn1** | **Beclin1** | Rn00586976_m1 |
| **Ckm** | **M-CK** | Rn01644605_m1 |
| **Ckmt2** | **miCK** | Rn01493732_m1 |
| **Cox4i1** | **COX IV** | Rn00567950_m1 |
| **Cpt1b** | **CPT-I** | Rn00682395_m1 |
| **Dnm1l** | **Drp1** | Rn00586466_m1 |
| **Esrra** | **ERRα** | Rn00433142_m1 |
| **Fis1** |  | Rn01480911_m1 |
| **Gabpa** | **Nrf2** | Rn01767215_m1 |
| **Gpx1** |  | Rn00577994_g1 |
| **Hk2** |  | Rn00562457_m1 |
| **Mfn1** |  | Rn00594496_m1 |
| **Mfn2** |  | Rn00500120_m1 |
| **MT-CO1** | **COX1** | Rn03296721_s1 |
| **Mybbp1a** |  | Rn00581478_m1 |
| **Myh7** | **β-MHC** | Rn00568328_m1 |
| **Nrf1** |  | Rn01455958_m1 |
| **Opa1** |  | Rn00592200_m1 |
| **Pdk4** |  | Rn00585577_m1 |
| **Ppara** | **PPARα** | Rn00566193_m1 |
| **Ppard** | **PPARβ/δ** | Rn00565707_m1 |
| **Ppargc1a** | **PGC-1α** | Rn00580241_m1 |
| **Ppargc1b** | **PGC-1β** | Rn00598552_m1 |
| **Pprc1** | **PRC** | Rn01411002_m1 |
| **Prkaa1** | **AMPKα1** | Rn00569558_m1 |
| **Prkaa2** | **AMPKα2** | Rn00576935_m1 |
| **Rcan1** | **MCIP1** | Rn00596606_m1 |
| **Sirt1** |  | Rn01428093_m1 |
| **Slc2a1** | **GLUT1** | Rn01417099_m1 |
| **Slc2a4** | **GLUT4** | Rn00562597_m1 |
| **Sod2** |  | Rn00566942_g1 |
| **Tfam** |  | Rn00580051_m1 |
| **Tfb1m** |  | Rn00710690_m1 |
| **Tfb2m** |  | Rn01412504_m1 |
| **Ucp3** |  | Rn00565874_m1 |
