# Supplementary material for: Resveratrol Improves Survival, Hemodynamics and Energetics in a Rat Model of Hypertension Leading to Heart Failure
Source: PLoS One. 2011 Oct 18;6(10):e26391. doi: 10.1371/journal.pone.0026391 (PMC3196575; doi:10.1371/journal.pone.0026391)
Supplement: Methods S1 — Supplementary Methods and Results. (DOC) [file pone.0026391.s007.doc]

**Supplementary Methods**

**Real-time quantitative PCR**

Frozen tissue samples were weighed, homogenized (Bertin Precellys 24) in ice-cold buffer. Total ventricular RNA was extracted using standard procedure with Trizol reagent (Invitrogen). cDNA were synthesized from 2μg total RNA according to the protocol provided with the High Capacity cDNA Reverse Transcription Kit (Applied Biosystems, France). Real-time PCR was performed using TaqMan Low Density Array (TLDA) technology. Pre-designed TaqMan probe and primer sets for target genes were chosen from an on-line catalog (Applied Biosystems, France). TLDA were designed to amplify 48 cDNA for each sample as follows: 43 target genes involved in energy metabolism and mitochondrial function and 5 housekeeping genes (Table S2). Each PCR reaction was performed on 4ng of cDNA in a volume of 1µl. The thermal cycling conditions were 2min at 50°C and 10min at 95°C, followed by 40 cycles of 15s at 95°C and 1min at 60°C. Quantification was achieved using the ΔΔC_t_ method. The average C_t_ obtained in LS group was used as a calibrator and the geometric mean of 14-3-3 protein zeta/delta (Ywhaz) and ribosomal protein P2 (Rplp2) housekeeping genes was used as the reference for normalization. The amplification efficiency of each probe is estimated maximal as described by the manufacturer.

Quantification of ANF (forward 5’–GGGCTCCTTCTCCATCACCAA-3’, reverse 5’-CTTCATCGGTCTGCTCGCTCA-3’), and BNP (GCTCTCAAAGGACCAAGGC, AACAACCTCAGCCCGTCAC) mRNA was assessed using the SYBR®Green method on a LightCycler rapid thermal cycler (Roche Diagnostics) as previously described [1]. For each target gene a standard curve was constructed from the analysis of a fivefold cDNA serial dilution and used for samples concentration calculation. Ywhaz (AGACGGAAGGTGCTGAGAAA, GAAGCATTGGGGATCAAGAA) and Rplp2 (GCTGTGGCTGTTTCTGCTG, ATGTCGTCATCCGACTCCTC) were used as housekeeping genes as their expression did not differ between groups. Geometric mean of their expression was used for normalization.

**Cardiomyocytes cultures**

Adult rat ventricular myocytes (ARVM) were dissociated by retrograde perfusion of healthy isolated heart with collagenase as described previously [2] with slight modifications. Freshly isolated cells were plated on laminin-coated culture dishes at a density of 3x10^5^ cells/dish in minimal essential medium (M4780, Sigma) supplemented with 2.5% fetal bovine serum, penicillin (100units/mL), streptomycin (100µg/mL), and 2% HEPES (pH 7.4) for 1 hour, and switched to serum-free medium for 20 hours. They were then incubated for 4 or 48 hours with resveratrol (RSV, 10µM) or its solvent ethanol 0.2% (control). Six independent cultures were used for each condition.

**Immunoblotting**

Frozen tissue samples or ARVM were homogenized (Bertin Precellys 24) in ice-cold buffer (50mg/ml). AMPK and ACC phosphorylation was assessed from cardiac tissue or cells protein extracts loaded onto 12% SDS-polyacrylamide gel. Blot was first split, part of which was incubated with a specific antibody for phospho-AMPK or total AMPK while the other half-blot was incubated with a specific antibody for phospho-ACC or total ACC. Anti-rabbit IgG, goat peroxidase-linked antibody was always used as secondary antibody. All antibodies were purchased from Cell Signaling Technology, Inc., Beverly, MA, USA.

For SERCA2, calsequestrin and eNOS, protein extracts from hearts or aortas were separated on 8% SDS-polyacrylamide gels. Blots were incubated with antibodies against SERCA2 (Santa Cruz Biotechnology Inc., Santa Cruz, CA, USA), calsequestrin (Affinity Bioreagents, Inc., Golden, CO, USA), and eNOS (Cell Signaling Technology). Values were normalized to protein loading evidenced by Coomassie blue.

Revelation was performed using an enhanced chemoluminescent substrate (SuperSignal West Dura, Pierce Biotechnology). Chemiluminescence was detected and quantified using a chemiluminescent detection system (ChemiDoc XRS, Bio-Rad) and image-analysis software (Quantity One, Bio-Rad).

**References**

1. Rimbaud S, Sanchez H, Garnier A, Fortin D, Bigard X et al (2009) Stimulus specific changes of energy metabolism in hypertrophied heart. J Mol Cell Cardiol 46: 952-959.

2. Verde I, Vandecasteele G, Lezoualc'h F, Fischmeister R (1999) Characterization of the cyclic nucleotide phosphodiesterase subtypes involved in the regulation of the L-type Ca2+ current in rat ventricular myocytes. Br J Pharmacol 127: 65-74.
